# Unraveling of Advances in 3D-Printed Polymer-Based Bone Scaffolds

**DOI:** 10.3390/polym14030566

**Published:** 2022-01-30

**Authors:** Yuanhang Xu, Feiyang Zhang, Weijie Zhai, Shujie Cheng, Jinghua Li, Yi Wang

**Affiliations:** 1 Basic Research Key Laboratory of General Surgery for Digital Medicine, Affiliated Hospital of Hebei University, Baoding 071000, China; 15227105095@163.com (Y.X.); 15176877696@163.com (F.Z.); zhaiweijie0924@163.com (W.Z.); chengshuj@126.com (S.C.); 2 National United Engineering Laboratory for Advanced Bearing Tribology, Henan University of Science and Technology, Luoyang 471000, China; 3 Department of Mechanical Engineering, Tsinghua University, Beijing 100084, China

**Keywords:** 3D printing, bone tissue engineering scaffolds, bone healing, polymer

## Abstract

The repair of large-area irregular bone defects is one of the complex problems in orthopedic clinical treatment. The bone repair scaffolds currently studied include electrospun membrane, hydrogel, bone cement, 3D printed bone tissue scaffolds, etc., among which 3D printed polymer-based scaffolds Bone scaffolds are the most promising for clinical applications. This is because 3D printing is modeled based on the im-aging results of actual bone defects so that the printed scaffolds can perfectly fit the bone defect, and the printed components can be adjusted to promote Osteogenesis. This review introduces a variety of 3D printing technologies and bone healing processes, reviews previous studies on the characteristics of commonly used natural or synthetic polymers, and clinical applications of 3D printed bone tissue scaffolds, analyzes and elaborates the characteristics of ideal bone tissue scaffolds, from t he progress of 3D printing bone tissue scaffolds were summarized in many aspects. The challenges and potential prospects in this direction were discussed.

## 1. Introduction

Bone is capable of self-healing, but cannot regenerate in special cases such as large defects due to the lack of growth and differentiation platform of bone repair-related cells [1,2]. At present, the options of treatments for critical-sized bone defects include autologous bone graft [3,4], allogenic bone graft [5,6,7], and artificial bone graft substitutes (i.e., bone tissue engineering scaffolds, BTES) [8,9]. However, auto-transplantation and allotransplantation will lead to secondary injury, poor size-matching, immune response, and other risks, limiting their clinical application [10,11,12]. To solve the above-mentioned problems of clinical treatment process, BTES with optimal biocompatibility and strong osteoinduction ability have been widely studied, especially the polymer-based composite bone scaffolds fabricated by 3D printing according to the clinical needs of patients, 3D printing technology can prepare accurately-controlled personalized-implants upon composition and structure, realizing the superior structure-function relationship and highly bioactive BTES, this cannot be accomplished by traditional processing strategy.

Recently, increasing attention has been paid to the research of polymer-based BTES, especially through the combination of natural polymers and synthetic polymer materials to create new tissue engineering scaffolds [13,14,15,16]. Bone tissue is composed of water, organic matter, and inorganic salt. The ideal BTES needs to simulate the original bone tissue structure. Natural polymers are biocompatible and biodegradable, but their mechanical strength and thermal stability are poor. The synthetic polymers possess ideal mechanical properties, but the hydrophobic surface leads to poor osseointegration [17]. For example, gelatin can simulate the biological properties of natural bone matrix protein, but it shows weak mechanical strength. The tissue scaffolds prepared by electrospinning combined with polylactic acid (PLA) have good biological and mechanical properties [18]. 

This paper reviews the research progress of polymer materials and tissue engineering scaffolds prepared by 3D printing technology. The properties of ideal BTES are summarized by introducing the process of bone healing, with emphasis on the polymer materials commonly used in 3D printing technology, the design optimization of scaffold structure, and its application in clinical medicine. In addition, it will describe the technical challenges arising from the current research and the potential prospects of 3D printed polymer BTES.

## 2. Process, Advantages, and Disadvantages of 3D Printing Technology

Natural bone consists of highly dense outer cortical bone and relatively loose inner cancellous bone. There is a Havers tube rich in blood vessels and nerves longitudinally in the cortical bone, and the vessels in the Havers tube are connected to each other in a transversely oriented channel, the Volkmann tube [19]. Cancellous bone is a reticular structure composed of plate or rod structure about 200 microns thick. 80% of the bone remodeling process occurs in cancellous bone [20]. Both types of bone undergo dy-namic remodeling, maturation, differentiation and absorption, which are controlled by the interaction between osteoblasts, osteoblasts and osteoclasts [21]. At the same time, extracellular matrix (ECM) provides mechanical support and appropriate environment for cell attachment, proliferation and differentiation. BTES are usually designed to simulate ECM to promote tissue regeneration. At present, a variety of manufacturing technologies are used to construct BTES, such as Solvent Cast-ing/Particulate Leaching [22], Gas Foaming [23], Freeze-Drying [24], Phase Separation [25,26], Electrospinning [27,28]. Sometimes the two techniques are used in combina-tion [29,30,31]. Among them, BTES fabricated by computer-aided design (CAD) modeling 3D printing technology possess the highest accuracy and repeatability as well as high spatial control ability scaffold upon microstructure, therefore it is one of the most ideal scaffolds for clinical application.

According to the material and manufacturing process, the typical printing methods of BTES can be divided into laser-assisted printing and non-laser-assisted printing. Laser-assisted printing technologies include stereo-lithography (SLA), selective laser sintering (SLS), laser-assisted Bioprinting (LAB). On the other hand, non-laser-assisted printing technologies, such as extrusion bioprinting, inkjet bioprinting and fused deposition modeling, have also been frequently reported in the literatures. This overview explains 3D printing methods in accordance with the above categories.

### 2.1. Laser-Assisted Printing

Given that there are many types of 3D printing processes, understanding the advantages and disadvantages of 3D printing processes and related materials will enable designers to make better decisions when choosing 3D printing processes to design and manufacture the best bone engineering scaffolds. Here are three types of printing grouped together.Because they all involve laser-assisted printing. Instead, the other three are classified as non-laser-assisted printing methods.

#### 2.1.1. SLA

**Figure 1:**
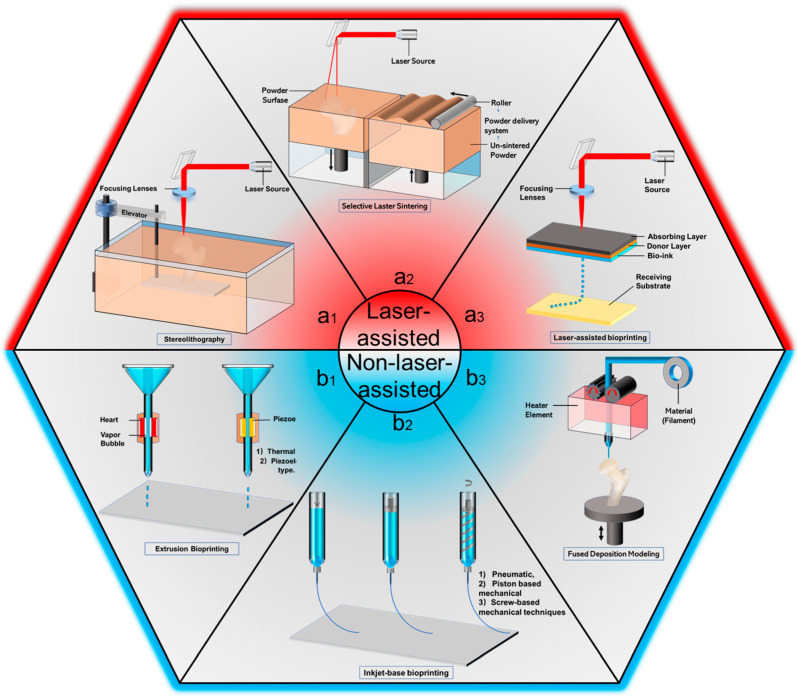
3D printing process diagram: (**a_1_**) SLS; (**a_2_**) SLA; (**a_3_**) LAD; (**b_1_**) Extrusion Bioprinting; (**b_2_**) Inkjet Bioprinting; (**b_3_**) FDM.

As a rapid prototyping process, SLA is one of the earliest 3D printing technologies used in BTES, which can be traced back to 1986. SLA solidifies the 3D scaffold through a polymerization process. Usually, a polymerization chain reaction is initiated on a layer or monomer solution by ultraviolet light or an electron beam. Once the first layer is fully cured, the platform is lowered a short distance in the vertical direction (Z-direction) and continues to connect to the new layer. These steps are repeated until a new model is completed (Figure 1(a_1_)). The opposite approach (layer-below-layer) is the most common approach for SLA, as it allows to finely control the stratification thickness.The platform is immersed in a photopolymer liquid and exposed to the focused light according to the desired design, where the polymer photopolymerizes. The non-exposed polymer remains liquid, after the cured layer is photopolymerized, the platform connected to the cured layer is lowered vertically so that another layer of unpolymerized liquid resin is applied to the top until the 3D bone scaffold structure is completed layer by layer. 

The most prominent advantage of SLA is the ability to print bone scaffolds of complex shapes at extremely high resolution, allowing the design of complex structures ranging in size from submicrons to decimeters. Compared with the minimum resolution of 0.005–0.010 mm for FDM and 0.060 mm–0.150 mm for SLA, the minimum resolution of SLA can reach 0.002 mm [32]. Although advanced scaffolds can be created using SLA, we acknowledge that there are some limitations in the application of SLA in scaffold preparation. First, because the polymer solution required for this type of printing is very sensitive to ultraviolet light, the choice of printing materials is minimal. Although the number of available polymer materials continues to increase, the technology is still limited to using only one polymer material at a time [33]. Second, uncured resins and residual photoinitiators may be cytotoxic and have poor biocompatibility [34]. Also, the manufacturing speed of SLA is relatively slow compared to other printing technologies, and the cost is relatively high.

#### 2.1.2. SLS

SLS is a powder-based additive manufacturing technology that creates 3D printed BTES with complex shapes by curing powdered materials layer by layer. Solidification is achieved by selective fusion or sintering of specified areas in each layer using the heat energy of the focused laser radiation system, as shown in Figure 1(a_2_). First, a layer of powder is deposited into the construction chamber. According to the cross-section data of the 3D CAD model, the laser beam sinters or melts the selective region on the powder layer to form a solid layer. Once the initial layer is complete, the construction platform is lowered by 100 to 200 microns, and a new layer of powder is deposited evenly and tightly onto the platform through a barrel rolled in the powder transfer system. A new layer of powder is then laser-sintered and bonded to the previous layer. This process is repeated until a complete 3D bone tissue engineering scaffold is created. Similar to SLS processing powders, Selective laser melting (SLM) and electron beam melting (EBM) are relatively new and rapid technologies.

The main advantage of SLS for bone tissue engineering is the 3D printing of biometals (e.g., titanium) and ceramics (e.g., hydroxyapatite). However, polymer materials such as polyether ether ketone (PEEK) with superior mechanical properties and biocompatibility are increasingly being used to manufacture bone scaffolds through SLS. Because of the high energy used in SLS to fuse the powder particles into a solid structure similar to natural cancellous bone, the scaffold shows high osteoblast attachment and bone differentiation [35,36]. The main limitation of SLS lies in the selection of polymer materials, the high temperature involved in the printing of scaffolds limits the use of cells and biological materials like FDM. The SLS printing method’s disadvantages are the energy consumption and high cost in the sintering process of SLS manufacturing bracket.

#### 2.1.3. LAB

LAB is a derivative application of direct-write technology and laser-induced forward transfer technology. A typical laser-assisted biological printer consists of a laser pulse, a material donor, and a receiving substrate, as shown in Figure 1(a_3_). The donor has a ribbon structure consisting of an energy-absorbing layer at the top (such as titanium or gold), a bio-ink layer at the bottom (such as cells [37] and polymers), and a Donor Layer in the middle. During the printing process, the energy absorbing layer generates pressure by receiving energy from the laser, then transmitted to the Donor Layer. The pressure is passed to the Donor Layer, which then uses the pressure to push the cell-containing material from the initial print material to the receiving substrate.

LAB has better cell printing resolution and accuracy than other bioprinting technologies and is one of the most attractive tools for in-situ printing of bone substitutes [38]. In addition, Lab does not face the problems associated with nozzles. First, when the diameter of the nozzle is minimal or the extrusion pressure is considerable, the shear stress of the nozzle will not cause the problem of inducing cell damage and death. Second, when the viscosity of biological ink is larger or the concentration of cells increases, it will not cause nozzle blockage.

### 2.2. Non-Laser-Assisted Printing

#### 2.2.1. Extrusion Bioprinting

Compared with inkjet bioprinting, continuous bio-ink lines can give a better whole-body interface to the generated 3D structure. The two main distribution mechanisms of bioprinting are pneumatic-based and mechanical-based. As shown in Figure 1(b_1_). In the printing process, a continuous force driven by either pneumatic pressure or piston or screw pressure extrudes an uninterrupted line of bio-ink through a micro nozzle, rather than a droplet. The extrusion material solidifies on the substrate and acts as a supporting structure. Next, the platform is lowered horizontally and another layer of bio-ink is added until the complete 3D structure is formed.

One of the most significant advantages of this technology over thermal-based or piezoelectric-based inkjet bioprinting is that it does not involve heating or piezoelectric processes, so it is easy to combine cells with bioactive substances. Second, with a few tweaks to the technology, The platform can squeeze bio-inks and continuously deposit various bio-inks. Switching between different containers to quickly manufacture complex structures [39]. However, the nozzle’s moving speed and the bio-ink viscosity’s nonlinearity affect the resolution of the extruded bioprinting [40]. Extruding bioprinting inevitably causes the shear stress of the print needle on the cells, which can also cause cell damage and death [41]. In the process of extruding bio-printing, the greater the extrusion pressure on the needle, the greater the shear stress on the cells in the bio-ink, and the lower the activity of the cells in the printing model [42]. Shao et al. Use ultrasound to assist the bioprinting process, which can alleviate the problem of reduced cell survival caused by blocked nozzles [43].

#### 2.2.2. Inkjet Bioprinting

Inkjet bioprinting is one of the most traditional methods used in nanomaterials. Although it works on the same principle as a traditional drip on-demand printer, biomaterial such as cells [37] and polymers replace the ink inside the cartridge, while a computer-controlled receiving substrate replaces the paper. Inkjet bioprinting is a common bioprinting technology based on the traditional inkjet printing method, which uses the desktop inkjet printer for bioprinting. This is a 3D printing method based on non-contact liquid drops, which uses thermal or compressed power to spray biological ink droplets from the nozzle of the printing head onto the hydrogel matrix or petri dish under the control of the computer, as shown in Figure 1(b_2_). In thermal-sensitive technology, air bubbles generated by local heating in an inkjet printer create a pulse of pressure that forces a drop of bio-ink from the print head to the substrate. In piezoelectric technology, In piezoelectric technology, a piezoelectric transducer generates pulses to generate enough pressure to eject droplets from the nozzle. Thermological or piezoelectric pressures are the two typical extrusions of the print head, which are basically controlled by custom CAD files to load the ink material onto the receiving substrate accurately. The 3D positioning system consists of a receiving substrate and two degrees of freedom (X and Y axis) print head.

The advantage of inkjet bioprinting is that cells and other growth factors (bio-ink) can be added to the printed polymer material, improving bone induction and bone conduction to overcome the limitations of conventional treatment options [42,44]. Although the shear and thermal stress of the nozzle on the cells during printing can affect the viability of the cells in the bio-ink, it has been shown in the literature that various cell types using inkjet bio-printing can produce 80–95% cell viability [45]. Secondly, its printing process is a non-contact process in the form of liquid droplets, from the nozzle to the receiving plate, the nozzle is completely separated from the printing material and the printing bracket. Compared with extrusion-based bio-printing, this printing technology can prevent the movement of the nozzle on the X-Y axis from interfering with the printing process. However, inkjet bio-printing also has some shortcomings and shortcomings [46]. The biggest drawback is the clogging of the nozzle, which makes it difficult to achieve smooth printing. There are two main reasons for the blockage of the nozzle: one is the viscosity of the polymer solution is too high, the other is that there are too many cells in the polymer solution [45]. Of course, in order to reduce the impact of this limitation, Gao et al. in the process of printing PEG peptide scaffold, the photopolymerization of acrylate esterified peptide and acrylate esterified polyethylene glycol (PEG) hydrogel co-printing method can minimize the problem of nozzle blockage to the greatest extent [47]. Furthermore, cell density, printing speed, and nozzle size in bio-inks are some known factors that affect the resolution and mechanical properties of inkjet bioprinting structures.

#### 2.2.3. FDM

As shown in Figure 1(b_3_), the continuous filament of thermoplastic material is heated in the heating element, melted into a semi-liquid state, and then extruded onto the building platform or the previously printed layer when the platform is lowered vertically. The process takes place layer by layer, with each layer deposited and fused together. After that, the printed 3D structure solidifies at room temperature, creating complex three-dimensional geometry. The complex three-dimensional geometry produced therein can be precisely managed by the motion of the manufacturing platform (in the Z direction) and the nozzle (in the X-Y direction) controlled by a CAD data file.

Compared with other printing techniques, FDM has many advantages in manufacturing functional parts of complex structures. By optimizing printing parameters including printing temperature, printing speed and printing layer thickness, Wang et al. adjusted the mechanical properties, surface quality and microstructure of the printing model to meet practical needs [48,49]. Although FDM has the advantages of low cost, fast speed, and easy process operation, FDM printing technology has limitations [50,51]. Biologics and natural polymers have limited applications in FDM due to the high temperature required for melting during FDM printing. The viscosity of the molten polymers limits the resolution of the printing that can be achieved, so you can only produce biological scaffolds with a fixed shape and relatively regular structure. In our view, much effort is needed to overcome these limitations.

## 3. The Process of Bone Healing

**Figure 2:**
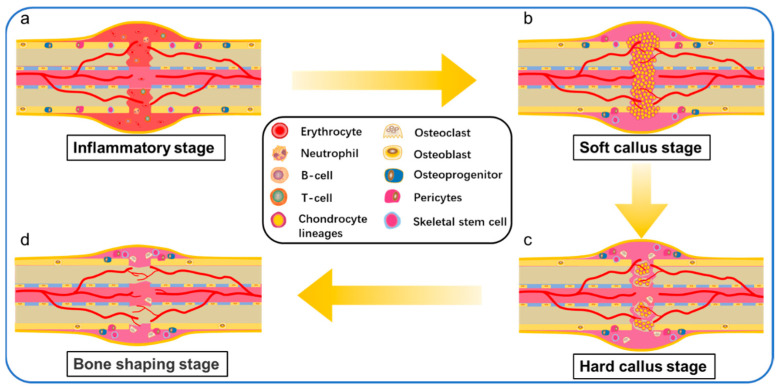
Repair processes of bone defeat. (**a**) Inflammatory stage; (**b**) Soft callus stage; (**c**) Hard callus stage; (**d**) Bone shaping stage.

Although fracture repair may not require support materials other than implants, bone grafts or bone substitutes may be required for bone defects exceeding the critical size. 3D printed biomaterial scaffolds with suitable interconnected porous structures play an essential role in bone tissue engineering. Especially for bone defects exceeding the critical size, they can not regenerate themselves. A matrix is needed as a scaffold to guide the activities of bone regeneration cells. The healing process of bone defects exceeding the critical size is a complex physiological process. Such bone defects of unstable long bone fractures are usually repaired by intramembrane ossification (IO) and endochondral ossification (EO) [52]. Repair initiates after the inflammatory reaction and includes a soft and hard callus formation stage. In the stage of inflammation, the rupture of blood vessels around the fracture end, the outflow of pulp through the rupture of bone marrow cavity, and the dilatation of capillaries of local inflammatory reaction resulted in the formation of hematomas around the fracture, and the osteocytes, periosteal cells and soft tissue cells around the broken end were necrotic (Figure 2a). The important sources of hematomas are neutrophils, macrophages, and platelets, which are involved in initiating inflammatory responses. Inflammatory cells are activated, triggering a cascade of reactions triggered by the hematoma, which, characterized by hypoxia and low pH, acts as a temporary scaffold for active invasion by local tissue macrophages and polymorphonuclear neutrophilsBone defects above the critical size lack similar temporary scaffolds, and bone implants (autologous, allogeneic, tissue-engineered scaffolds, etc.) are required to perform this role.

After the hematoma was mechanized into granulation tissue, there were a lot of new blood vessels at the fracture end, and osteoclasts continued to remove the remaining dead bone. The initial stage of repair starts from the distal site of the stable and good peripheral vascular cortical bone fracture, from the release of the inner layer of periosteum cells continuously concentrated, proliferated, and differentiated into braided bone. Chondroblasts began to appear in the periosteum near the fracture end and formed chondroid tissue to replace the granulation tissue, leading to the formation of the bone collar through IO (Figure 2b). At this stage, early vascularization plays an important role in healing bone defects. Because early vascularization is the basis of blood supply to the bone defect site, slow or incomplete vascularization will lead to insufficient oxygen and nutrient supply, resulting in hypoxia and cell death of the implanted material cells, or preventing the growth of host bone cells. As Ronald e. Unger, bone tissue engineering scaffold need not only bone cell growth, proliferation and differentiation in the biological material, and must happen quickly the new vascularization and graft internal blood flow [53]. In the stage of cartilage eschar formation, around the fracture space, the mechanical strength of the fracture is unstable and the local microenvironment hypoxia caused by the interruption of blood supply leads to the formation of cartilage mass (Figure 2c). The chondrocytes gradually mature into hypertrophic chondrocytes (HCs). HCs secret collagen X in the transition zone where cartilage will be replaced by bone tissue and expresses a variety of active molecules, promoting mineralization (Figure 2d) and vascular invasion into the distal fracture site of the callus and reorganizing the outer boundary of the periosteum of the callus. At this stage, the arrangement of bone trabeculae is loose and irregular. 

In the stage of hard eschar formation, the whole cartilage was constantly replaced by woven bone, the irregular new bone trabeculae in the original callus were gradually thickened, the arrangement began to be regular and dense, and the Harvard system of cortical bone was re-established. Osteoclasts and osteoblasts invaded the dead bone at the fracture end to complete the crawling replacement process of dead bone clearance and new bone formation. The original eschar was replaced by lamellar bone, so that the fracture site formed a strong bone connection. Then the pulp cavity recommunicates and finally forms the bone frame through the fracture space. In forming new bone links by self stress, bone scaffolds made of metal [54] and ceramics [55,56] often encounter a problem—stress barrier. Because metals and ceramics have high elastic modulus, when they are implanted into bone defects as bone scaffolds, their long-term contact with bone will reduce the physical load on the surrounding bone, resulting in the decrease of bone mineral density around the bone scaffolds and even secondary damage.

In contrast, the elastic modulus of polymer-based composite scaffolds can be adjusted according to their own ratio [57,58]. The bone scaffolds are sufficient to provide mechanical support for weight-bearing bone construction before newly formed bone synthesis and can not be too high to avoid stress shielding. Different cells and signaling pathways participate in the process of bone repair, and various cells and signaling pathways play a coordinator role. During the differentiation of osteoblasts, the activation of transcription factors at a specific time provides necessary clues to specify the function of bone progenitor cells when cells transform into osteoblasts. Ankit salhotra et al. Discussed the interaction between transcription factors (such as SOX9, Runx2, OSX and activated transcription factor 4 (ATF4)) necessary for osteoblast differentiation [59]. More and more researchers load these transcription factors and primordial progenitor cells into bone tissue engineering scaffolds, so that when bone tissue engineering scaffolds are implanted in vivo, they can quickly activate the proliferation and differentiation of primordial bone cells. For example, it has been confirmed that the application of bioactive proteins such as bone morphogenetic protein (BMP)—2 and basic fibroblast growth factor (bFGF) is an effective way to improve the bone inducibility of bone scaffolds. Ren et al. delivered BMP-2 and bFGF to poly (L-lactic-co-glycolic)/graphene oxide/hydroxyapatite nanofibrous scaffolds. It was found that BMP-2 can induce the differentiation of bone cells, BFGF promotes the proliferation of bone cells, which makes the nanofiber scaffold have excellent bone induction rate and regeneration activity [60]. Because of the excellent performance of BMP-2 in inducing osteocyte differentiation, Zhang et al. coupled derived bone morphogenetic protein 2 derived from bone anoligopeptide (ssvpt, Ser ser ser Val Pro THR) with dopamine coating on 3D printed polylactic acid (PLA) scaffolds. He also established a rat skull defect model to verify that the scaffold has high bone conductivity for the adhesion and proliferation of rat bone marrow mesenchymal stem cells (MSCs) [61].

3D printing technology can adjust the pore diameter, porosity and microporous structure of bone scaffold according to the parameters in the printing process, and customize the shape of a matched bone defect. However, 3D printing structures made of single biomaterials (such as PLA, PLGA, etc.) lack the biological activity required by the ideal requirements for inducing bone formation. It is necessary to add additives to promote bone induction and bone transmission in scaffolds. These additives are more than natural factors and cell involvement in bone healing, so exploring the mechanism of bone healing plays a vital role in the preparation of 3D printed bone tissue engineering scaffolds.

## 4. Properties of Ideal Bone Scaffolds

BTES used to repair bone defects should possess several important characteristics: biocompatibility, biodegradability, mechanical properties, controllable structure. Zhang et al. demonstrated BETS prepared by natural and synthetic polymers as a promising method for creating novel tissue-engineered scaffolds. Because the scaffolds combine the advantages of the two materials and meet various requirements, including biological activity, mechanical properties, controllable degradability, and other properties [62].

### 4.1. Biocompatibility

Biocompatibility is the primary criterion for all tissue-engineered scaffolds. First, BTES should not inhibit the activity of bone tissue cells, that is, allow cell adhesion, migration and proliferation. Second, BETS should neither show any significant cytotoxicity during or after transplantation nor induce a positive immune response to prevent severe inflammation. Printable and biocompatible polymer materials are the optimum material in the applications of 3D printing. Because they possess high adjustability and complexity and provide a prefer bionic environment for living cells [63]. Therefore, more and more people have begun to commit to the research of biological materials needed for 3D printing in recent years, especially in polymer-based composite print materials.

### 4.2. Biodegradability

Controllable biodegradability is also a special property realized by scaffolds. Biodegradation of implanted materials will inevitably affect the mechanical support of growing bone tissue. They provide a biological and mechanical framework for the growth and differentiation of cells. It is eventually replaced by regenerated tissue designed to match the mechanical properties of natural bone. Ideally, the rate of scaffold degradation synchronizes with the rate of mineralized tissue deposition. The gradual reduction of mechanical support provided by the degradable scaffold is compensated by the gradual increase of mechanical support provided by the new tissue. If the scaffold degrades too quickly, the scaffold will not be able to provide mechanical support while new bone is formed, which is likely to exceed the load and may lead to fracture. Conversely, if the scaffold does not degrade quickly, it can trigger an inflammatory response to foreign substances in the scaffold, thus preventing tissue regeneration. In order to prepare scaffolds that can both enhance the activity of osteoblasts and display appropriate degradation rates, there have been many studies on the interaction between degradation and osteoblasts in vitro [64]. The biodegradability of polymers is an attractive property that can be controlled by the molecular design of polymers.

### 4.3. Mechanical Properties

As the essential properties of bone grafts, mechanical properties (i.e., elastic modulus and compressive strength) are the main challenges for applying 3D printing technology to porous scaffolds for bone tissue engineering. Many factors affect the mechanical properties of BTES, such as the properties of biomaterials and pore structure. On the one hand, the mechanical properties of most polymer BTES are low, but the optimized post-treatment method and component modification can improve the mechanical properties of polymer scaffolds. On the other hand, the pore density and size of the scaffold significantly affect the growth and adhesion of cells. However, the porosity is inversely proportional to the mechanical properties, so the mechanical properties of multi-pore BTES sometimes cannot meet the stress of natural bone. In addition, the mechanical properties of bone tissue engineering materials had better match that of natural bone to avoid the stress shielding phenomenon. This phenomenon usually occurs in the application of traditional metal bone scaffolds. Suppose the elastic modulus of the scaffold is much greater than the elastic modulus of the surrounding tissue. In that case, most of the stress will be borne by the scaffold (mostly metal scaffold) rather than the surrounding bone.

Over time, mechanical stimulation of bone cells around the scaffold is reduced, ultimately leading to decreased bone density (osteopenia) in healthy bone tissue near the scaffold [65], accelerated absorption of surrounding bone, and even secondary infection. In general, the mechanical properties of the scaffold should be sufficient to provide mechanical support for weight-bearing bone construction before the synthesis of newly formed bone, but not too high to avoid stress shielding.

### 4.4. Microstructures

The microstructure of scaffolds is also critical in promoting cell viability and tissue growth. In the absence of an engineered blood supply, the interconnected pore structure allows for the inward diffusion of oxygen and nutrients and the outward diffusion of waste from the scaffold. Besides, porosity also supports cell migration into the scaffold and increases the available surface for cells to bind to the scaffold and interact with the surrounding tissue. In the design stage of BTES, the microstructure of the scaffold must be precisely designed with parameters favorable to cells and tissues, including the porosity of the scaffold, the pore size and the interconnected pore structure, etc.

#### 4.4.1. Porosity

It is well known that the skeletal structure of adult bones is not uniform and radially graded, consisting of two distinct structural regions. The outer high-density areas are called cortical bone with a porosity of 5% to 30% (mostly in the 5% to 10% range), while the inner areas are called cancellous bone with a porosity of 50% to 90%. Considering the gradient structural characteristics associated with natural bone porosity, the design of regenerative bone scaffolds can simulate the porosity changes between cortical and cancellous bone to promote regional cell differentiation. Given the radiating gradients of natural bone, Andrea et al. have designed a method that can be used in combination with human mesenchymal stem cells (HMSC). The porosity of each scaffold area is similar to the gradient found in natural bone, with 29.6% ± 5% porosity in the outer ring and 50.8% ± 8.1% and 77.6% ± 3.2% porosity in the central and inner regions, respectively. They also matched the size of the scaffold to the structural characteristics of the natural bone. This porous scaffold with a gradient structure of 500 μm in the outer ring, 750 μm in the middle, and 1000 μm in the inner. Cell differentiation was confirmed by up-regulated gene expression of Runx2 and bone sialoprotein markers. The experiment showed that optimizing the porosity and pore size of BTES by imitating the natural bone structure was beneficial to the differentiation of HMSC and the mineralization of bone tissue [66].

#### 4.4.2. Pore Size

The size of the individual pore size within the scaffold is an important consideration. Small pores (a few microns to tens of microns) promote cell adhesion, intracellular signal transmission, cell proliferation and migration, while large pores (a few hundred microns) facilitate angiogenesis, ECM aggregation, and tissue formation. The pore size of a BTES depends mainly on the anatomical location and type of bone tissue (e.g., cortical or trabecular) in the human body. Diao et al. showed that the pore size design of bone tissue engineering porous scaffolds should consider the type of bone defect. He studied three β-tricalcium phosphate scaffolds with different pore sizes (100, 250, and 400 µm). Then, he found that 100 µm pore size scaffolds enhanced osteoblast differentiation during intramembranous ossification. It is more effective in inducing bone formation and is most suitable for repairing flat bone defects. The 400 µm scaffold can accelerate the formation of cartilage template and ossification center in endochondral ossification, showing the best ability of bone formation to repair extended bone defects. Numerous studies have demonstrated the importance of the pore size of scaffolds in bone engineering and indicated that the pore size of scaffolds should generally be between 100 and 300 μm to allow cell penetration, migration, and growth, and to achieve optimal tissue vascularization. However, there is no clear consensus on the effect of optimal pore size on optimal mechanical and osteogenic properties [62].

#### 4.4.3. Pore Structure

In addition to the porosity and pore size affecting the microstructure of the scaffold, the interconnected pore structure within the scaffold also plays a crucial role in influencing its function. As more and more people study the pore structure, the pore structure of different scaffolds tends to be diversified.

**Figure 3:**
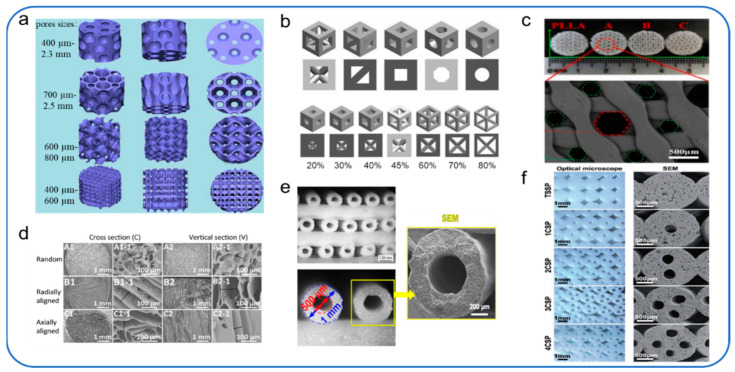
Pore structure diversity of 3D printed bone scaffolds. (**a**) Based on the observation and analysis of lotus root structure and bone trabecular structure, four kinds of porous scaffolds were proposed; reproduced with permission from Zhipeng, H. et al. [66], published by Composites Science and Technology 2020. (**b**) Five pore geometries were selected (triangular prism with a rounded and a flat profile, cube, octagonal prism, sphere) and seven porosities (up to 80%), on the basis of which 70 models were created for finite element analyses;reproduced with permission from Piotr, P. et al. [68], published by Materials 2020; (**c**) The porous g-HNTs/g-MgOs/PLLA composite scaffolds with large and small pores and honeycomb structure; reproduced with permission from Kun, L. et al. [69], published by Composites Part B: Engineering 2020; (**d**) SF/COL composite scaffolds with random pores, radially aligned pores or axially aligned pores; reproduced with permission from Xue, F. et al. [70], published by Journal of Materials Chemistry B 2020; (**e**,**f**) Hollow channels structure; reproduced with permission from Wenjie, Z. et al. [71], published by Biomaterials 2017; reproduced with permission from Chun, F. et al. [72], published by Advanced Science 2017.

Piotr et al. studied 3D-printed porous bone tissue scaffolds based on shape memory polymer composites (SMPC). The microstructure of the scaffold is based on the observation and analysis of the bone trabecular structure of the lotus root. Four different pore shapes (circular holes, polygonal holes, randomly oriented holes) are designed and supported (Figure 3a). The reliability of the structures was demonstrated by mechanical experiments and micromechanical theoretical studies, while biological experiments verified the biological activity and osteogenic effect of the scaffolds. The results showed that the round hole and polygonal hole scaffolds were more beneficial to promote early adhesion, proliferation, and osteogenesis. Although the scaffolds with random orientations and directional orientations showed good cell adhesion, no significant cell proliferation was observed early. In the later observation species, the scaffolds with random orientations could promote cell proliferation and osteogenesis [67].

Piotr et al. selected five pore geometries (triangular prism with circular and flat contours, cube, octagonal prism, Sphere) and seven porosity (up to 80%), and 70 models were constructed for analysis (Figure 3b), to select the appropriate pore geometry and scaffold porosity for orthopedic regenerative medicine. On the one hand, the researchers placed the scaffold in the flow channel to estimate the growth media velocity and wall shear stress. On the other hand, the researchers placed scaffolds in the bone to assess osteoblastic proliferation. The results of this study evaluated the effects of different pore shapes and porosity of scaffolds on bone regeneration. They provided a basis for the appropriate selection of pore geometry and porosity of scaffolds in orthopedic regenerative medicine [68].

In order to solve the problems of poor mechanical properties and limited osteogenic activity of scaffolds in the past, Liu et al. developed a novel 3D printed composite scaffold consisting of poly-lactide (PLLA) matrix, surface-grafted MgO whiskers (g-MgOs) and Helosite nanotubes (g-HNTs). The scaffold not only combines the printability of PLLA, the excellent osteogenic activity of g-MgOs, and the excellent enhancement and toughening effect of g-HNTs, but also optimizes the microstructure of the scaffold. Based on the consideration of cell proliferation and migration, the researchers designed a scaffold combined with a large (510 ± 20 μm) and a small (210 ± 15 μm) honeycomb (Figure 3c), with a porosity of 74.15 ± 5.32%. The experimental results show that the interconnecting pore structure in the scaffold makes the media have favorable fluidity, which is conducive to the entry of nutrients, the proliferation and migration of cells [69].

Optimizing the pore structure of scaffolds can influence the regeneration of bone defects of critical size and the remodeling process and regeneration of osteochondral defects. Considering the influence of different pore structures on the regeneration of osteochondral defects in vivo, Feng et al. prepared silk fibroin/collagen (SF/COL) composite scaffolds with three pore structures (random pore, radial pore and axial pore) (Figure 3d) to evaluate the effect of pore structure on the remodeling process and regeneration of osteochondral defect tissue. The results showed that the scaffolds with radial and axial pore arrangement had better regeneration ability and endogenous repairability than those with random pore arrangement, especially those with radial pore arrangement [70].

Vascular formation in bone scaffolds plays an important role in bone regeneration, but the vascularization of bone regeneration is slow in the critical size of bone defects. It is well known that blood vessels are initially formed by endothelial cells organized into microtubules. According to previous studies, it was found that a microfluidic system composed of a group of microchannels can be used to induce endothelial cells to form incomplete blood vessels in vitro [73]. Zhang et al. designed a kind of silicate bioceramics (BRT-H) bone scaffold with hollow tube structure and bioactive ions by optimizing the microstructure parameters of the 3D-printed bone scaffold (Figure 3e). The scaffold utilizes the synergistic effect of the conduit structure of the BRT scaffold and the bioactive ion composition to solve the problem of slow vascularization of large bone regeneration. The results showed that the hollow tube structure facilitated the transfer of stem cells and growth factors and possessed a synergistic effect with bioactive ion products in enhancing the regeneration of vascularized bone [71]. Considering that microchannel structure can induce endothelial cells to form a basic vascular system, Feng et al. were inspired by the microstructure of the natural plant lotus root. A bionic scaffold with a multi-channel structure (Figure 3f) was successfully prepared by optimizing the microstructure parameters of the 3D-printed bone scaffold. The results showed that the scaffold improved the porosity and specific surface area and significantly improved the adhesion and proliferation of BMSCs in vitro, as well as osteogenesis and angiogenesis in vivo compared with traditional 3D printing materials [72]. Both of the above two papers have demonstrated that optimizing the microstructure of bone scaffolds is conducive to cell transport and regeneration (blood vessels and bone) within the scaffold. Although many 3D-printed scaffolds with different pore structures have been developed, the optimal pore structures affecting bone growth are still being studied.

In conclusion, despite these latest studies, there is still much to be learned about the effect of designed scaffolds on bone regeneration in future in vivo studies. Thus, it remains a challenge to successfully balance scaffold properties conducive to cell function, cell viability, and mechanical integrity under load-bearing conditions.

## 5. Polymer Material of 3D-Printed Bone Scaffolds

Polymers offer greater design flexibility than metals and ceramics. Biopolymers have received significant attention in material development due to their extensive availability, low toxicity/non-toxicity, biodegradability, biocompatibility, chemical versatility, and inherent functionality. The biocompatibility of biological materials affects the functional properties of 3D-printed tissues and organs. Cells need to be attached to the surface of the implanted biomaterial to maintain its activity and proliferation to promote tissue regeneration. Therefore, the selection of biocompatible materials is critical to the formulation design of biological inks. In this section, we present the fundamental aspects of these different biopolymers that can be linked to their processing and material applications; below is the summary table (Table 1).

### 5.1. Natural Polymers and Mixtures Based on Natural Polymers

Natural polymers are considered as a class of macromolecules with multiple monomers or compound monomers linked together to form long chains. They are usually identical or very similar macromolecules that are the building blocks of extracellular tissue. As a result, there are few problems with foreign body reactions when they are implanted as implants. In addition, they usually perform biological functions at the molecular level. Most commonly used in biomaterials, natural biopolymers include chitin and chitosan, alginic acid, collagen, gelatin, hyaluronic acid, and fibrinogen.

#### 5.1.1. Chitin and Chitosan

Chitin is the second most abundant carbohydrate after cellulose. It is a polysaccharide found in crustaceans (exoskeleton of crab and shrimp shells and cell walls of fungi and yeast). Its derivative, chitosan, is obtained by deacetylation of chitin. Chitin and its deacetylated derivative chitosan are natural polymers composed of randomly distributed β-(1-4)-linked d-glucosamine (deacetylated unit) and n-acetyl-d-glucosamine (acetylated unit) [93]. N-acetylglucosamine exists in the composition of chitin and chitosan, so a common degradation product is n-glucosamine. However, n-glucosamine, a substance naturally present in the extracellular matrix of eukaryotic cells, is non-toxic to the human body and biocompatible when applied to bone scaffolds. N-Acetylglucosamine (GlcNAc) is a monosaccharide that is typically polymerized linearly through the (1,4)-β bond [94]. It is also because the components of chitin and chitosan both have n-acetylglucosamine, which can accelerate tissue repair and prevent the formation of scar tissue. However, in actual application, when the traditional chitin/chitosan lacks the necessary mechanical strength, the mechanical properties of the composite scaffold can be optimized by blending chitin/chitosan with other materials [95]. Deepthi et al. reviewed that many studies have adopted different methods to incorporate hydroxyapatite (HAP), bioglass ceramic (BGC), silicon dioxide (SiO_2_), titanium dioxide (TiO_2_), and zirconium oxide (ZrO_2_) into chitin or chitosan scaffolds to enhance the mechanical properties of the scaffolds themselves [96]. One of the most important manifestations of the biocompatibility of chitin/chitosan scaffolds is their ability to promote cell adhesion, proliferation, and differentiation [97]. Studies have shown that chitin/chitosan composite scaffolds have a good affinity for bone marrow mesenchymal stem cells and osteogenic induction capacity, and have broad application prospects in bone regeneration. The research results showed that the content of chitosan in the composite nanofibers could affect the growth of bone marrow mesenchymal stem cells. He placed rat bone marrow mesenchymal stem cells (rBMSCs) on nanofiber membranes with three different concentrations of chitosan. After seven days of culture in vitro, the nanofiber membrane with the mass ratio of chitosan to PCL of 30:70 showed the strongest proliferation effect on BMSCs compared with the nanofiber membrane with the mass ratio of chitosan to PCL of 50:50 and pure PCL nanofiber membrane. On the 14th day, the expression levels of osteogenic genes Runx2, ALP, and OCN reached the highest in the group with a chitosan/PCL mass ratio of 50:50 nanofiber membrane. On day 21, the ratio played the most significant role in promoting calcium deposition. The results showed that chitosan could promote the proliferation and osteogenic differentiation of rBMSCs, and its activity on the chitosan nanofiber scaffold was affected by such factors as composition, composition ratio, and action time [74].

Although chitin and chitosan have good biocompatibility, biodegradability, antibacterial activity, non-antigenicity, and high adsorption performance, there are still many problems and challenges in the clinical application of chitin/chitosan nanofibers. We can improve the preparation process of the chitin/chitosan bone scaffold to make it more suitable for the biomedical application of bone regeneration.

#### 5.1.2. Alginate

Sodium alginate is a natural biopolymer consisting of two monosaccharide units, namely a-L-mannuronic acid (M unit) and b-D-guluronic acid (G unit) linked by 1,4-glycosidic bond and composed of different GGGMM segments. Alginate tends to gel with divalent cations under normal physiological conditions, one of the most important properties apart from biocompatibility and biodegradability. Alginate is widely used in the formulation of extrudable mixtures for 3D printing due to its ability to increase the viscosity of polymer solutions.

Although interpenetrating polymer network (IPN) hydrogels composed of gelatin and hydroxypropyl cellulose (HPC) have been prepared by continuous enzymatic and chemical crosslinking methods, such hydrogels have better mechanical properties than networks composed of two or more interpenetrating polymers [98]. Bone tissue engineering scaffold requires high mechanical properties, and interpenetrating hydrogel will inevitably reduce polymer concentration and increase the water content. Therefore, Luo et al. prepared an alginate/gelatin interpenetrating scaffold with uniform nano-apatite coating through 3D printing and in-situ mineralization [75]. The scaffold realizes double crosslinking of alginate and gelatin through CaCl2 and EDC solution so that the scaffold has good mechanical property and good biological activity. In addition, the uniformly distributed nano-apatite coating was prepared by 3D printing and in-situ mineralization technology on this scaffold. The uniformly distributed nano-apatite coating enhanced the adsorption of protein on the surface of the scaffold and significantly promoted the proliferation and osteogenic differentiation of rBMSCs [99].

#### 5.1.3. Collagen

Bone is composed of cells embedded in the ECM, which is an ordered network composed of two major nano-phases: collagen fibers composed of type I collagen molecules and hydroxyapatite nanocrystals distributed along the collagen fibers [21]. A collagen is a group of at least 29 different polymeric proteins that are the most abundant protein component of the ECM. ECM provides a special physiological microenvironment for cells, protects cells from harmful mechanical effects, and mediates mechanically-induced signal transmission. Actually, the bone scaffold that promotes bone regeneration functions as an ECM. All collagen proteins are in a triple helix structure consisting of hydrogen bonds, and the main amino acid groups include glycine, proline, and hydroxyproline. The fibrils formed by these triple helix structures are strong and highly flexible and can be further crosslinked to improve the mechanical properties of different types of collagen [100]. However, different types of collagen have rather complex and diverse structures, splice variants, additional non-helical domains, assemblies, and functions. Among them, collagen fibers of type I and type V are involved in the structural skeleton of the bone, while collagen fibers of type II and type XI are mainly involved in the fibrous matrix of articular cartilage [101]. Their torsional stability and tensile strength determine the stability and integrity of these structures. Therefore, bone scaffolds based on collagen have been widely used to treat bone/cartilage defects and bone/cartilage regeneration. Bone scaffolds composed of type I collagen usually rely on the sclerosis of type I collagen through calcium phosphate mineralization and cross-linking with substances such as HAP.

#### 5.1.4. Gelatin

Gelatin is a mixture of polypeptide and protein obtained by partial hydrolysis of collagen extracted from animal skin and bone [102]. It widely exists in mammals and has excellent biological activity, such as cell adhesion, biocompatibility, and biodegradability [103]. Moreover, it does not have antigenicity under a Physiological environment [104]. It is widely used in various biomedical applications, and currently, it is often used in clinical wound dressings and adhesives. In bone tissue engineering, gelatin in injectable form can be used as a delivery carrier for cells. There are also a large number of studies on the mixing of gelatin with different polymers. Because of its suitability and biocompatibility, gelatin has been extensively studied in the field of bone regeneration by mixing with nano-HAP [105], β-tricalcium phosphate [106], and chitosan [107].

However, gelatin can form a thermoreversible gel with water. Gelatin shows a gel state at low temperatures (below 25 °C) and a dissolved state at high temperatures (above 35 °C) [108]. Gelatin is very sensitive to process temperature due to its sol-gel transformation [109]. Therefore, it is challenging to use FDM technology for the 3D printing of gelatin. It is necessary to modify the surface of the gelatin. There are amine groups, carboxyl groups, and other pendant groups in the gelatin molecule, which can be used as active sites for gelatin modification. Wang et al. mixed gelatin with bacterial cellulose (BC) treated with tempo-medicated oxidation (TO-BC) and maleic acid (MA-BC). The carboxyl group in cellulose can be cross-linked with the amide in gelatin through the hydrogen bond interaction between N-Hydroxysuccinimide (NHS) and 1-(3-dimethyl aminopropyl)-3-(ethyl carbodiimide hydrochloride) (EDC) to enhance the mechanical strength and gel stability of gelatin, which is more suitable for printing of tissue scaffolds [35].

To determine the mechanical strength difference of gelatin, studies have found that glutaraldehyde (GTA) reacts with the base of the polypeptide chain to form Schiff bases, which can stabilize the gelatin structure through further reaction with other glutaraldehyde molecules during the formation of crosslinking [110]. Kathleen et al. found that gelatin scaffolds crosslinked with GTA showed enhanced mechanical properties and stability. Meanwhile, the cytotoxicity of glutaraldehyde to cells can be neutralized by lysine [111].

#### 5.1.5. Hyaluronic Acid

Hyaluronic acid (HA) is an acidic, non-sulfated glycosaminoglycan consisting of D-glucuronide and N-acetylglucosamine disaccharide units. As a component of the extracellular matrix, HA can retain water in tissues, support cellular structures and act as a lubricant because its hydroxyl groups can tightly bind water molecules to chains through hydrogen bonds. HA is a major component of the ECM of articular cartilage (AC). HA maintains the normal homeostasis of cartilage sites by regulating cell function, including promoting the phenotype of chondrogenic genes, as well as the production and retention of matrix components [112].

HA interacts with cells through the surface receptor CD44 and regulates cell movement and adhesion [113]. HA also helps to reduce the immunogenicity of the embedded cells as this biocompatible material reduces the adsorption of protein [114]. Therefore, it has attracted significant attention in the biomedical fields, such as bone regeneration therapy, wound healing, and drug delivery. Despite these excellent biocompatibility properties, HA is an ideal biomaterial for cartilage tissue engineering, but it lacks the mechanical properties required for application to three-dimensional extrusion-based bioprinting (EBB). Limited by this reason, the uniform distribution of cells cannot be ensured. HA also lacks the gelling ability necessary to maintain the 3D structure after the printing process. Cristina Antich et al. performed 3D printing with HA and PLA and the resulting scaffold showed good mechanical properties, including printability, good gel strength, and degradation [115].

#### 5.1.6. Cellulose

Cellulose is composed of β-D-glucopyranose covalently linked by C1 (carbon atom) and C4 (β-1,4-glycosidic bond) through acetal interaction [116]. Cellulose has the advantage of low cost and high elasticity [117]. As a major component of plants, cellulose is a sustainable and nearly inexhaustible polymeric feedstock [118], and cellulose has many derivatives, including cellulose ethers/esters, micro/nano-sized cellulose products, etc. Many cellulose derivatives such as cellulose ether and microcrystalline cellulose (MCC) have been important commercial products for many years [119]. Cellulose can be used as a matrix with a natural layered network and porous structure and contain abundant functional groups (especially -OH group) on the surface of cellulose fiber, which can be combined with other materials.

Cellulose and its derivatives are materials suitable for 3D printing. Since cellulose cannot be melt processed, identification/development of a good solvent for cellulose is essential for its utilization. However, cellulose is insoluble in water and ordinary organic solvents due to the formation of intramolecular and intermolecular hydrogen bonds [120]. To date, only a few solvent systems have been able to dissolve cellulose. Generally speaking, cellulose solvents can be divided into derivative solvents and non-derivative solvents [121]. Under the action of derivative solvent, cellulose hydroxyl undergoes yellowing, esterification, etherification, and other functionalization reactions. All these will destroy the intramolecular and intermolecular hydrogen bonds between cellulose molecules and lead to the dissolution of cellulose. Non-derivatizing solvents, such as ionic liquids, can dissolve cellulose through physical molecular interactions without prior derivatization.

### 5.2. Synthetic Polymers and Mixtures Based on Synthetic Polymers

Natural polymers have good biocompatibility and biodegradability. However, their compression modulus did not reach the normal level for cancellous bone (>100 MPa) [121,122,123]. So they are mechanically weak and cannot withstand the forces exerted on the bone [124]. Therefore, they are mainly used as additives or composites because their bone-inducing properties and the ability to enhance cell and protein adhesion benefit from good bionic action [125]. Synthetic polymers are often used as 3D bone tissue engineering scaffold materials or composited with other materials in 3D printing technology due to their good mechanical properties. At present, the commonly used synthetic polymers suitable for 3D printing BTES mainly include PLA, polycaprolactone (PCL), and polycarbonate (PC), PEEK, Polypropylene (PP), Polyamide (PA), etc. Acrylonitrile butadiene styrene (ABS) can perfectly simulate natural bone in vitro and is often used in clinical model teach-ing aids or surgical models.Researchers can choose appropriate 3D printing technology to meet actual printing needs based on the properties of synthetic polymer materials and their advantages and disadvantages.

#### 5.2.1. Acrylonitrile Butadiene Styrene

Acrylonitrile butadiene styrene (ABS) is an economical and efficient polymer, simple to manufacture, it has good impact resistance, chemical resistance, processability, and suitable strength and characteristics [126]. ABS is a thermoplastic and amorphous polymer made of polymerized styrene, acrylonitrile, and polybutadiene. The melting temperature of ABS is 105 °C, making ABS a common candidate for FDM and SLA systems. In recent years, it has been found that the addition of montmorillonite (OMMT) can effectively improve the tensile strength of 3D printing ABS, which makes the composite polymer more excellent in mechanical properties and thermal stability, thus becoming a promising printing material in FDM [127]. ABS tissue scaffold can provide an appropriate environment for cell growth and matrix regeneration for cartilage and intervertebral disc repair, and it is usually used for bone tissue engineering such as cartilage [77]. most of the ABS is not biodegradable, but ABS M30i plastic has good degradability [128]. However, because the ABS material cannot pass through the autoclave [129], which is an important Preoperative procedure before implantation in the human body, the ABS tissue scaffold cannot be used in the human body. However, it can perfectly simulate natural bone in vitro and is often used in clinical model teaching aids or surgical models [130,131,132] to make detailed surgical plans and considerably shorten the operation time.

#### 5.2.2. Polylactic Acid

PLA refers to a series of polymers: Pure poly-L-Lactic acid (L-PLA), Pure poly-D-Lactic acid (D-PLA), and poly-D, L-lactic acid (DL-PLA), homopolymers of L-PLA are mostly used in clinical practice. Each monomer can be different from other materials in the composition of copolymers suitable for 3D printing [133]. Unlike ABS, PLA has the advantages of low cost, biocompatibility, and biodegradability [134,135]. The melting temperature of PLA is between 173 and 178 °C, so it is relatively easy to be used to make scaffolds in the temperature range of 190–230 °C [136], which is why PLA is widely used in FDM. PLA still has good mechanical properties and has a similar compressive strength (230 MPa) as bone [137], making it suitable for musculoskeletal tissue engineering. However, PLA has many disadvantages. First, the tensile strength of pure PLA materials is poor, while stretchability is very important in FDM technology because they affect the extrusion of material filaments and layer stacking. Bartolomeo et al. printed the PLA and clay composite at a higher printing temperature, and the clay enhanced the thermal stability of PLA and remarkably enhanced its elastic properties [138]. Secondly, the release of lactic acid by-products in the degradation process need to be noticed [139]. In the early stage of degradation, lactic acid can be metabolized by itself. In the late stage of degradation, many lactic acid by-products can form a local acidic environment, leading to tissue inflammation and cell death. To address this problem, calcium phosphate may be used as a buffer to maintain the PH of the internal environment at 7.4 [140].

#### 5.2.3. Polycaprolactone

Similar to PLA, PCL is also a kind of low-cost and degradable polyester. The melting temperature of PCL is 58–60 °C [141], and it has excellent elastic properties [142]. The tensile strength of block PCL is 10.5~16.1 MPa, the modulus is 343.9~364.3 MPa, and the tensile yield strength is 8.20~10.1 MPa [143]. Compared with many polyester materials, PCL is more suitable for scaffold construction by FDM. In SLS printing, Shaun Eshraghi et al. tested the PCL samples and found that the mechanical properties would decrease sharply with the increase of porosity. However, the low-stress region could be designed as the void region by adjusting the SLS parameters, thereby improving the mechanical strength of PCL and manufacturing the tissue scaffold with controllable mechanical properties [143].

The PCL can degrade for a long time [144] and still exist after being placed in organisms for 12 weeks [145], which can provide long-term protection and support for tissue healing and regeneration and does not form potentially harmful byproducts like PLA. Muwan Chen et al. embedded hyaluronic acid, methylated collagen, and terpolymer into PCL’s pore-like structure, and a good cell seeding rate was achieved over a period of time [76]. In summary, by selecting the appropriate printing method and combining with the long-term degradation rate of PCL, we can make scaffolds with both composite stress requirements and controllable degradation in different parts.

#### 5.2.4. Polycarbonate

Polycarbonate (PC) is a common high-strength chemical material, able to withstand temperatures below 140 °C without physical deformation. Common PC materials are synthesized by the chemical industry, while some researchers try to recover the plastic part of electronic waste products from obtaining PC materials, thus reducing carbon dioxide emissions during the process of synthesizing PC materials. After the recovered PC material was subjected to 3D printing, an intensity close to that of a commercial PC printing material was obtained [81]. But with the depletion of chemical fuels such as petroleum and their biological toxicity [82], a popular way to synthesize PC in the field of 3D printing implants in the human body is to polymerize isosorbide extracted from corn starch.

A bio-based PC, which is polymerized by isosorbide extract from corn starch, not only has excellent tensile properties suitable for 3D printing but no bio-toxicity has been observed [83,84].

Seong et al. produced four samples of ABS, PLA, chemically synthesized PC, and bio-based PC using 3D printing technology, respectively. The bio-based PC showed the highest tensile strength, elongation at break, and elastic modulus among the four materials. It was also determined that the bio-based PC had good rheological properties at the optimum temperature in the range of 240 °C to 270 °C [146]. However, the PC easily absorbed moisture from the air. Many small defects were formed on the surface of the material when the moisture evaporated, which affected the performance and printability. Seong et al. overcame this defect by performing at 80 °C for 4 h [146].

#### 5.2.5. Polyetheretherketone

Polyetheretherketone (PEEK) is a member of the polyaryletherketone polymer family, and its chemical structure can maintain resonance stability, making it extremely inactive [147]. PEEK has low water absorption [148,149] and can be maintained in an aqueous environment for long periods. It has been found in the early research that the rigidity of PEEK did not change significantly after multiple continuous pressurized steam cycles [150]. Such high-temperature resistance enables high-temperature steam sterilization of PEEK material without affecting the material performance. Of course, due to its high-temperature resistance, it needs to be adjusted to a higher temperature for extrusion when 3D printing is performed.

PEEK has excellent mechanical properties, with its Young’s modulus of 3.6 GPa and tensile strength of 100 MPa [151]. Studies have made composite printing of PEEK with different contents with other materials, which can improve the mechanical strength of the printing scaffold and realize the specific customization of specific tissues in the human body [152]. In the past, metal materials were often used for tissue replacement due to the high-intensity movement of the mandible. However, due to the stress shielding effect, it was not conducive to the patient’s recovery. Because PEEK has similar mechanical strength to human bones, desirable results were observed in mandibular replacements [85,153].

In addition, it is worth mentioning that PEEK has a high transmittance, which enables doctors to more clearly observe the local reactions and tumor recurrence after surgery through CT or MRI, giving it unique advantages over traditional metal implants in the field of bone tumors (such as spinal tumors) [154].

#### 5.2.6. Polypropylene

Polypropylene (PP) is a crystalline (crystallinity 60–70%) thermoplastic polymer with the advantages of low cost, strong versatility, excellent physical, mechanical, and thermal properties, and its melting point is 165 °C [87].

PP is one of the lightest polymers in common printing materials (density: 0.908 g/cm ^− 3^). The yield strength (32 MPa) and stiffness of PP are slightly lower than those of ABS or PLA, but with longer service life [88]. These two characteristics make PP material suitable for replacing tissue scaffolds which require lightweight and low-stress requirements. The mechanical strength of PP can be enhanced by mixing PP with other materials. However, although PP has a wide range of chemical compatibility, its low surface energy results in that most materials cannot be easily adhered to its surface [89]. At the same time, studies have found that PP can shrink and curl due to crystallization in the printing process, which is not conducive to printing [90]. Because of the above two points, Darsani et al. grafted PP with maleic anhydride (MA) and found that it could improve the surface adhesion property of PP. At the same time, the blending of Palm oil fuel ash (POFA) and PP increased the adhesion of polymer materials and improved their shrinkage during printing [91].

#### 5.2.7. Polyamide

Polyamide (PA) is one of the commonly used polymers for tissue engineering applications. PA has good biocompatibility, mechanical properties, excellent wear resistance, and chemical stability. PAs are therefore commonly used in SLS technology for accurate printing and replacement of bone tissue, but can also be used in FDM technology [1].

In that SLS process, it is required that the material have a relatively low melt viscosity to achieve the desired melt flow rate without inputting too much energy while reducing the loss of the driving device [155], PA is a semi-crystalline polymer with good sintering properties and relatively low melt viscosity, in particular PA12, which is a very suitable material for SLS technology [156].

In recent years, there have been many studies on the preparation of composite materials with bioactive materials such as PA and HAP to replace bone tissue scaffolds [92]. The addition of bioactive materials such as HA increases the composite material’s tensile strength, stiffness, and thermal stability. Although the addition of these materials increases the friction of the composite material and reduces the content of PA, the study has found that the melt flow rate of the composite material is not much different from the common ABS material [157], and can still be used as one of the appropriate materials for 3D printing.

## 6. 3D-Printing for Medical Applications

3D printing technology can print the bone model of real patients and print bone substitutes and scaffolds to promote bone repair.

### 6.1. Bone Model

The internal organ model made by 3D printing technology [158] has the same anatomical and geometric characteristics as the natural organ. This transparency allows the surgeon to see anatomical structures such as blood vessels and lymphatics associated with parenchyma [159]. Because of the accuracy of the anatomy of the model, this is equivalent to providing a “surgical road map”. The accurate visualization of the complex relationship between anatomical structures can more easily identify and plan the operation [160], which is very important in the discussion of preoperative planning. Detailed preoperative planning can improve the success of the operation and reduce the overall risk to surrounding tissues and organs, as well as to patients. At the same time, it can also reduce the operation time, and may even improve the surgical results. This also means shorter hospitalization and lower medical costs.

**Figure 4:**
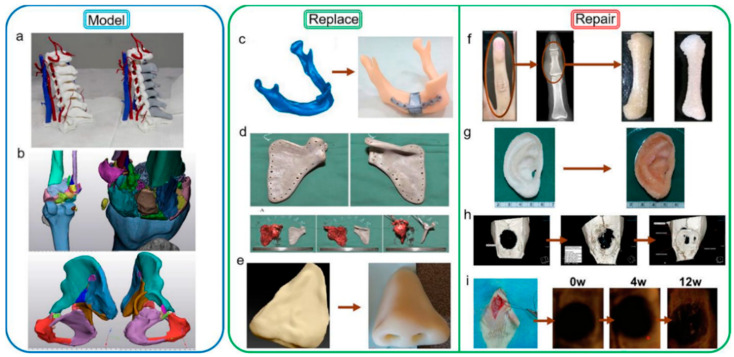
3D-printed for Medical Applications. (**a**) Preoperative full-color analysis model of a patient with C6 vertebral bone tumor; (**b**) Preoperative modeling of comminuted fractures in the knee and pelvis; (**c**) Gradient lattice mandibular prosthesis prepared by metal coated polymer; reproduced with permission from Xiao, R. [161], published by Composites Part B: Engineering 2020, 193, 108057; (**d**) Scapular prosthesis made with PEEK; reproduced with permission from Liu, D. [162], published by J Bone Oncol 2018, 12, 78–82; (**e**) Middle phalangeal bone implant prepared by sPLA/N-HAP composite; reproduced with permission from Nuseir, A. [78], published by J Prosthodont 2019, 28, 10–14; (**f**) Micro-ct showing BMP-2 loaded SFF stent promoting bone healing;reproduced with permission from Gupta, A. [163], published by ACS omega 2017, 2, 4039–4052; (**g**) human ear-shaped cartilage reconstruction based on tissue engineering scaffolds; reproduced with permission from Zhou, G. [164], published by EBioMedicine 2018, 28, 287–302; (**h**) Micro-CT images of bone regeneration in the defects of BMP-2-loaded SFF scaffold; reproduced with permission from Lee, J.W. [79], published by Biomaterials 2011, 32, 744–752; (**i**) Micro-CT images of bone regeneration in the defects of Biodegradable 3D scaffolds; reproduced with permission from Li, X. [165], published by Macromol Biosci 2018, 18, e1800068.

The Medical 3D Image Printing Center of the Affiliated Hospital of Hebei University successfully completed the research and development of the full-color preoperative analysis model of C6 vertebral bone tumors designed with CAD assistance in 2017. It is the first time in the field of 3D printing of cervical vertebrae that the technique of avoiding errors in the second phase of the arteriovenous phase is introduced. Operators can complete surgical navigation in the operating room through a tablet computer or mobile phone. Meanwhile, the posterior spinal canal structure can be printed as a model after CAD design (Figure 4a). It can completely open and observe the posterior structure of the vertebral body, which helps the operator to understand the lesion and surrounding situation more conveniently and intuitively. In 2020, the most complex model of comminuted fracture and the innovation of surgical planning was realized, such as the comminuted fracture model of knee joint and pelvis in Figure 4b, which enabled doctors to use the reconstructed model to talk and discuss with the family members of the patient before operation, so that the communication with the patient and his family was clearer, and the simple language description was no longer the same as in the past, which made the family members of the patient confused.

### 6.2. Bone Replacement

Trauma and tumors of bone tissue can lead to large bone defects. The mechanical strength of natural bone was restored by in-situ filling with tissue engineering scaffolds.

Patients with bony organ defects face great psychological stress because the absence of bony tissue such as the nose, mandible and scapula can make a face or body image less acceptable to the general public [166,167]. Therefore, the printing of bone substitutes is very necessary.

**Figure 5:**
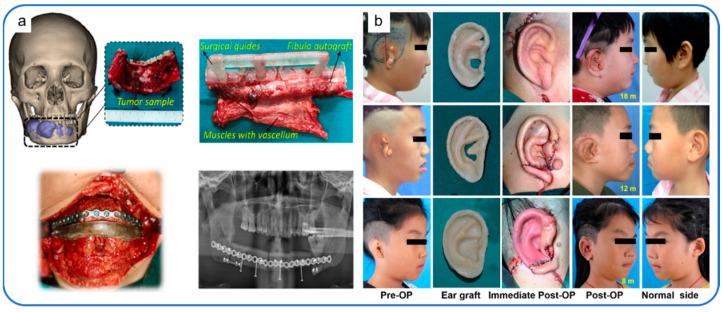
(**a**) The clinical implantation and post-operative effect of 3D-printed PEEK implant; reproduced with permission from Kang, J [86], published by J Mech Behav Biomed Mater 2021, 116, 104335; (**b**) Composite tissue scaffold loaded with cartilage cells implanted in the human body; reproduced with permission from Zhou, G. [178], published by EBioMedicine 2018, 28, 287–302.

Reconstruction of facial bones is particularly challenging in the field of in vitro bone implantation because of the important functional and aesthetic considerations of facial bones [168]. A mandibular defect can adversely affect the patient’s appearance, speech, chewing and social activities. Therefore, joint, occlusal, facial symmetry, donor area, vascular pedicle, soft tissue area and thickness should be considered in mandibular reconstruction [169]. Successful reconstruction requires the restoration of symmetrical appearance, sufficient masticatory space and correct joint position as much as possible, and the operation and control of bone position are required to be very high. The usual methods for mandibular defect repair usually include autograft or allograft [170,171] and metal graft [172]. In autologous and allogenic bone transplantation, most of the donor bone segments are straight (fibula, scapula, composite radius) or slightly curved (ilium crest) [173,174]. It is often necessary to have osteotomy on the donor bone, which causes secondary injury to the donor area and cannot fit the anatomical shape of the jaw perfectly [175]. Large differences in elastic modulus and relative density between metallic solids and human bones may lead to osteoporosis caused by stress shielding [172]. This problem can be solved by implanting the mandibular substitute shown in Figure 4c by 3D printing technology, and the density of the material can be increased in the high-stress area to match the human body. In the low-stress area, the density of the material is reduced, the weight of the implantation is reduced, and the waste of the material is reduced [161]. Kang et al. developed a BTES for mandibular defects combined with a 3D-printed polyetheretherketone (PEEK) implant and the free vascularized fibula graft [86]. Improved the stress barrier caused by the metallic materials commonly used in the past, and at the same time have sufficient mechanical strength to cope with the masticatory motion, as the picture shows, the bone tissue scaffold restores the patient’s mandibular anatomy to a nearly normal state (Figure 5a).

3D printing technology also has a good application in the bone repair of large joints. A benign fibrohistiocytic tumor of bone (BFH) is an invasive primary bone tumor. When the local resection is incomplete, the risk of recurrence is very high, so it needs to be completely resected. The main challenge for clinicians is bone reconstruction after tumor resection. 3D printing technology can help doctors solve this problem. Liu et al. report that PEEK replacement (Figure 4d) was performed in patients with benign fibrohistiocytoma of the scapula by 3D printing. The imaging examination of the patients after the operation showed that the substitute was in a good anatomical position. The patient’s shoulder joint activity was tested: 120° on the lift, 90° adduction, 50° on the external rotation, and 70° on internal rotation. the results of surgery are satisfactory [162].

A common tissue replacement for the face is also a nose prosthesis. The loss of a nose can be devastating for the affected person, as changes in facial features can be accompanied by a variety of psychological, functional and cosmetic difficulties [176]. Surgical reconstruction of such defects is usually limited by insufficient soft tissue, hard tissue residue, and vascular damage. Therefore, removable facial prostheses are an attractive and feasible alternative to help patients reintegrate into the community and resume their daily lives. Amjad et al. reported a case of nasal repair in which the nose was completely lost due to traffic accidents. Polyglycolic acid fibers were prepared into accurate human nasal alar shapes by three-dimensional printing technology [78] (Figure 4e). In addition, by coating with polylactic acid, the scaffold can obtain sufficient mechanical strength to maintain its original shape during cell culture until the ala cartilage shown in Figure 4e is finally formed. After testing, the tissue engineering scaffold has a faster bone formation rate and better rigidity than natural cartilage tissue (Young modulus = 8.60 ± 2.19 MPa) [177].

### 6.3. Bone Repair

BTES can load drugs, cytokines, and cells to promote in situ regeneration, so as to restore the normal anatomical structure of the human body.

In Arvind et al. research, the fibers with a diameter of about 1.6 mm were produced by melt treatment of the prepared biological composites by using a twin-screw micro extruder, and successfully used in 3D printing of representative middle finger bone (Figure 4f) [163].

Jin et al. developed a 3D scaffold with embedded drug delivery microspheres to improve the enhancing ability of honeybee cells in bone regeneration. He used BMP-2 growth factor as the releasable drug in the microspheres and successfully observed good regeneration of bone tissue, as shown in Micro-CT result (Figure 4h). It is also confirmed that the pore interconnection of bone tissue instead of scaffold is a critical factor in bone regeneration [79].

**Figure 6:**
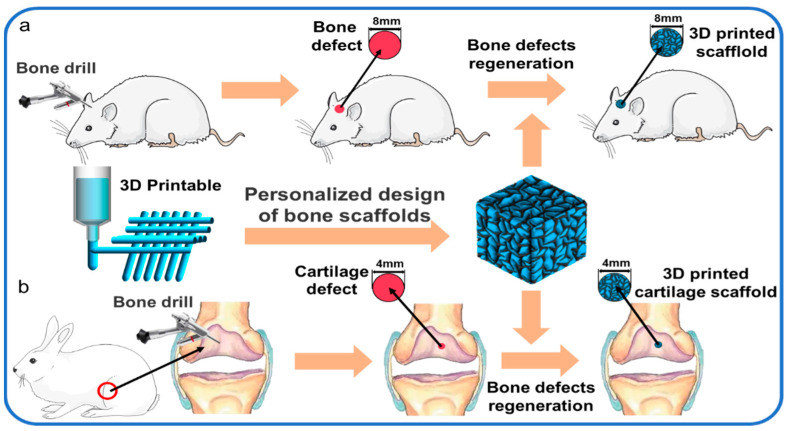
(**a**) regeneration of skull defect in rats; (**b**) articular cartilage regeneration in rabbits.

When the Large bone defect is caused by bone tissue trauma, it is believed that inhibiting the inflammation of the wound site is beneficial to accelerate the regeneration of the wounded tissue. Li et al. prepared a biodegradable three-dimensional scaffold composed of the polymer matrix (polylactic acid and polyethylene glycol), ceramics (nano-hydroxyapatite) and anti-inflammatory drugs (Dex). After the test, it was found that the scaffold has the ability to induce bone formation and anti-inflammatory ability, but because it is not loaded with growth factors or stem cells, its bone formation rate is slow (Figure 4i), and the material degradation rate is not ideal. However, this study proved that PLA composite scaffold can accelerate bone regeneration and can be used for future tissue engineering applications [165].We drew a rough flow chart to describe the experiment (Figure 6a).

Cartilage tissue has no blood vessels inside, and its nutrient supply depends on vascular transport in the perichondrium, which leads to its limited self-repair ability. The use of tissue engineering, that is, the technology of implanting polymer scaffolds of progenitor cells to produce new tissue equivalents, has been considered. It is a potential means to produce replacement cartilage. Cartilage cells or their precursors can generate new cartilage tissue, so cellular components are commonly used [80]. However, cartilage is a highly specialized connective tissue. Due to the nature of its composition and structure, its self-repairing ability is limited. Circumstances can lead to degeneration after injury. Sun et al. reported a study, they used 3D printing technology to make a hydrogel loaded with mesenchymal stem cells, using rabbits as a model for evaluation, and found that they achieved better cartilage production and anti-inflammatory effects than the natural environment in the living body [164]. We drew a rough flow chart to describe the experiment (Figure 6b). Zhou et al. reported the first clinical case of 3D printing material successfully implanted in the human body to treat microtia. They inoculated autologous auricle chondrocytes on a multi-component (PCL/PGA/PLA) scaffold for 3D printing(Figure 4g), which not only perfectly restored the external anatomy shown, but also observed the successful regeneration of the patient’s ear cartilage after the operation [178] (Figure 5b).

## 7. Conclusions

Three-dimensional (3D) printing makes it possible to manufacture any object highly precisely without causing any assembly or material waste. It is lighter than objects made by traditional methods and has the ability to meet the stress of different bone tissues in the human body. These implants or objects (extended into 3D objects) produced by 3D imaging are designed to match the patient’s specific anatomical structure accurately, and at the same time, they can also function as cells and drug carriers. However, there are still many problems to be solved in applying 3D printing technology in the human body.

1. In vitro drug release experiments cannot fully simulate the physiological conditions in vivo. There is no guarantee that the same drug release curve as in vitro experiments will be achieved in humans, and only certain time points can be selected for drug release concentration detection. Therefore, medical devices with the ability to monitor drug delivery efficiency in real-time should be valued by researchers. This device can be mounted on a 3D printed tissue engineering scaffold, enter the body together, release the drug through external equipment, and always check whether the drug concentration is maintained within the treatment range.

2. When there is a large area defect in bone tissue, the strength of the synthetic scaffold is mostly unable to meet the normal stress requirements in the human body. Therefore, many researchers strengthen the function of the scaffold by loading growth factors or stem cells in the tissue scaffold. However, heterologous cells and proteins may cause an immune response and cause health problems. Therefore, the 3D printed scaffold should be biocompatible, matching the natural bone stress without triggering an immune response. At the same time, stem cell therapy is more expensive, and cost reduction is necessary. In addition, the ethical issue of implantation in the body is another limitation in the whole process.

3. There are different types of 3D printing technologies on the market, each with its advantages and limitations. Traditional manufacturing methods print materials with rough surface texture and low overall strength. 3D printing uses accurate CAD models to achieve precise printing, and post-processing of implant stents manufactured by 3D technology can solve this problem, but it will increase the cost. So this technology is only suitable for customization, not mass production systems. The cost of the machine and the requirements of professional and technical personnel are also the main limitations of this technology. From this, it can be seen that how to make 3D printing maintain accuracy while carrying out assembly line production should be one of the focuses of future research.

4. Although the 3D printed scaffold has micro/nano-sized pores on the surface, it can be used as a carrier for cells, cytokines, drugs, and other substances. It can also play a role in transporting nutrients and supporting cell growth, but it cannot be controlled. The pore size and spatial distribution limit the application of 3D printed stents, which is something we should pay attention to.

## Figures and Tables

**Table 1:** Application of polymer materials, applicable processing technology, advantages and disadvantages.

Polmer Type	Application	AM Technique	Advantages	Disadvantages	Ref
Chitin and chitosan	Bone repair	Extrusion Bioprinting	Osteogenic induction	Lack of mechanical strength	[74]
			Biocompatibility		
Alginate	Bone repair	Extrusion Bioprinting	Biocompatibility	low cell adhesion and proliferation	[75]
			Improve polymer viscosity		
Collagen	Bone repair	FDM	Similar to the natural extracellular environment	Weak mechanical properties	[75,76]
		Extrusion Bioprinting			
		Inkjet Bioprinting		Fast degradation rate	
		Laser-Assisted Bioprinting			
Gelatin	Bone repair	Extrusion Bioprinting	Biocompatibility	Difficult to hot working	[35]
	Inkjet Bioprinting		Low antigenicity	Poor mechanical strength	
HA	Bone repair	FDM	Cell support	Poor mechanical strength	[76]
			lubrication		
Cellulose	Bone repair	Extrusion Bioprinting	low cost	Unable to melt	[35]
			High elastic	indissolvable	
ABS	Bone repair	FDM	low cost	Unable to autoclave	[77]
	Bone model	SLA	Chemical resistance	Poor degradability	
			Good workability		
PLA	Bone repair	FDM	low cost	Poor tensile strength	[78,79,80]
	Bone replacement		Biocompatibility	Harmful metabolite	
	Bone model		Degradable		
PCL	Bone repair	FDM	low cost	Poor mechanical strength	[76,80]
		SLS	Degradable	Long degradation time	
			High elastic		
PC	Bone repair	FDM	high intensity	Easy to absorb moisture from the air	[81,82,83,84]
			Non-biotoxicity		
			High tensile strength		
PEEK	Bone replacement	FDM	Low water imbibition	Processing requires a higher temperature	[85,86]
			High temperature resistance		
		SLS	high transmittance	biologically inert surface	
			excellent mechanical properties		
PP	Bone repair	FDM	Lower density	Lower rigidity	[87,88,89,90,91]
			Good workability		
			Easy to curl		
PA	Bone replacement	FDM	Biocompatibility	lack of shape stability	[92]
			Strong mechanical properties		
		SLS	Wear Resistance		
			Chemical stability		

## Abbreviations

BTES, bone tissue engineering scaffolds; CAD, Computed Aided Design; FDM, Fused Deposition Modelling; SLA, Stereolithography; SLS, Selective Laser Sintering; LAB, Laser-assisted Bioprinting; HA, hyaluronic acid; ABS, Acrylonitrile Butadiene Styrene; PLA, Polylactic Acid; PCL, Polycaprolactone; PC, Polycarbonate; PP, Polypropylene; PEEK, polyetheretherketone; PA, Polyamide.
